# Role of *Rhizomys pruinosus* as a natural animal host of *Penicillium marneffei* in Guangdong, China

**DOI:** 10.1111/1751-7915.12275

**Published:** 2015-03-30

**Authors:** Xiaowen Huang, Guohua He, Sha Lu, Yuheng Liang, Liyan Xi

**Affiliations:** 1 Key Laboratory of Malignant Tumor Gene Regulation and Target Therapy of Guangdong Higher Education Institutes, Research Center of Medicine, Sun Yat-sen Memorial Hospital, Sun Yat-sen UniversityGuangzhou, China; 2 Department of Dermatology and Venereology, Sun Yat-sen Memorial Hospital, Sun Yat-sen UniversityGuangzhou, China; 3 Department of Dermatology and Venereology, The Second People's Hospital of Liwan DistrictGuangzhou, China; 4 Guangdong Provincial Center for Disease Control and Prevention, Institute of Pathogenic MicrobiologyGuangzhou, China

## Abstract

*Penicillium marneffei*, a dimorphic fungus that can cause penicilliosis marneffei, is endemic in Southeast Asia. The only known hosts of *P. marneffei* are humans and bamboo rats. The aim of our study was to explore the distribution of *P. marneffei* in bamboo rats, their associated environment and non-rat-associated environments. Totally, 270 samples were collected in Guangdong province of China in 2012; the prevalence of *P. marneffei* was much higher in samples collected from surrounding areas of burrows (8.2%) than in the samples obtained from non-rat-associated sites (2%) or artificial farms of bamboo rats (0%). There was no difference in *P. marneffei* isolated rate from different areas of Guangdong province. The infection is prevalent in all rats, and this fungus could be frequently seen in the rats' lungs. This study confirms that bamboo rat is the ecological niche of *P. marneffei* and hypothesizes that bamboo rats become infected by inhaling aerosolized conidia originating from environmental sources, rather than by the fecal–oral route or transplacental crossing. According to the result of no detection of *P. marneffei* in the artificial farm, the activity of bamboo rats might be more relevant to the distribution and dissemination of *P. marneffei* in natural environment.

## Introduction

*Penicillium marneffei*, the only known dimorphic and pathogenic species in the genus of *Penicillium*, is an emerging pathogen that can cause lethal penicilliosis marneffei. This infection is endemic in Southeast Asia, including Thailand, Vietnam, Hong Kong, Southern China, Taiwan, India and Laos (Hu *et al*., 2013). In these endemic areas, penicilliosis marneffei has been proven to be the third commonest AIDS-indicating systemic opportunistic infection among HIV-positive patients (Wong and Wong, 2011). So far, more than 500 cases have been reported in Southern China (Hu *et al*., 2013).

Since the organism was discovered in 1956 from bamboo rats, it has been isolated from the internal organs of four species of rodents (*Rhizomys sinensis*, *Rhizomys pruinosus*, *Rhizomys sumatrensis* and *Cannomys badius*) (Chariyalertsak *et al*., 1996a; Fisher *et al*., 2004a; Gugnani *et al*., 2004; 2007; Vanittanakom *et al*., 2011). In the past few decades, several studies attempted to find out whether penicilliosis marneffei occurs as a consequence of zoonotic or sapronotic transmission. Till now, it reveals little association between bamboo rats and this infection epidemiologically. Studies in Thailand have shown that the infection rate of penicilliosis marneffei did not increase in the people who often had contact with bamboo rats. Despite this, the distribution of bamboo rat species generally followed the distribution of *P. marneffei* (Vanittanakom *et al*., 2011; Cao *et al*., 2011; Li *et al*., 2011,2011), and the multilocus microsatellite typing system showed little difference in allele frequencies between *P. marneffei* isolates from bamboo rats and human, which means these two host-associated populations of *P. marneffei* shared high similarity (Fisher *et al*., 2004b; Cao *et al*., 2011). On the other hand, soil exposure, especially during the rainy season, was found to be a risk factor associated with the infection caused by *P. marneffei* (Chariyalertsak *et al*., 1996b; 1997). So far, the available information seems to suggest that exposure to soil, rather than the enzootic reservoir, is the dependent factor that is associated with the increasing risk of *P. marneffei* infection. However, to date, the achievement of extensive efforts for recovering *P. marneffei* from environments remains limited. The definitive evidence of the environmental reservoir for *P. marneffei* within the soil or other substrates is still lacking.

We reviewed the cases of *P. marneffei* infection previously reported, and concluded that more than 82% of the cases were reported in Guangxi and Guangdong province of mainland China (Hu *et al*., 2013). Cao and colleagues (2011) and Li and colleagues (2011,2011) have shown that in these two provinces, the infection with high prevalence exhibits in bamboo rats of the genera of *Rhizomys* and *Cannomys*. Here, we want to identify the natural cycles of infection by *P. marneffei*, so we attempted to recover isolates of *P. marneffei* from rat burrows, rural regions and the artificial propagation farm of bamboo rats from six different districts across Guangdong province. This is the first study to investigate the existence of *P. marneffei* in bamboo rats, natural environment and artificial propagation farm.

## Results

### Prevalence of P. marneffei across Guangdong province of China

**Fig 1:**
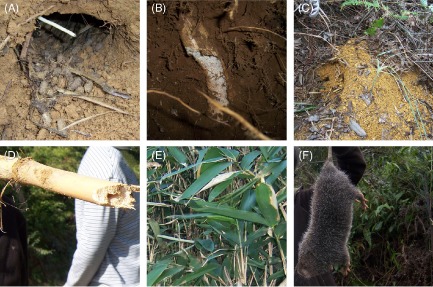
The bamboo rat (*R. pruinosus*) we captured and surrounding environmental samples. (A) Rat's cave and stool of the rat; (B) bamboo root in the burrow; (C) the soil and debris of food; (D) petiole; (E) bamboo leaves; (F) bamboo rat.

One hundred eighty-four samples were collected from the places near bamboo rats' burrows, and 50 soil samples were collected from the places far away from the burrow (with an average distance of 1500 m). From Fig. 1A–E, we could find the burrows excavated by bamboo rat and the surrounding areas, including the stool of bamboo rat, bamboo root in the burrow, the soil and bamboo leaves. Figure 1F showed the bamboo rat captured from the burrow. Among the collected 184 samples surrounding the burrow, 15 isolates of *P. marneffei* were recovered from 15 samples (Table 1). There was no statistical difference in the recovery of *P. marneffei* among diverse regions in Guangdong province (*P* > 0.05). In the obtained 50 soil samples far away from the burrows, only one sample was positive for *P. marneffei*. All these isolates were further identified by DNA sequencing of the ITS region.

**Table 1:** *Penicillium marneffei* recovered from different districts of Guangdong province

Collecting districts	No. examined	No. positive for *P. marneffei*
Chaozhou^a^	23	4
b	10	0
Qingyuan^a^	28	2
b	10	0
Zhaoqing^a^	34	4
b	10	1
Meizhou^a^	44	3
b	10	0
Shaoguan^a^	55	2
b	10	0
Artificial farms	36	0
Total	270	16

a Samples were collected from the places near bamboo rats' burrows.

b Samples were collected from the places far away from the burrow.

### The isolation of P. marneffei from environment

**Fig 2:**
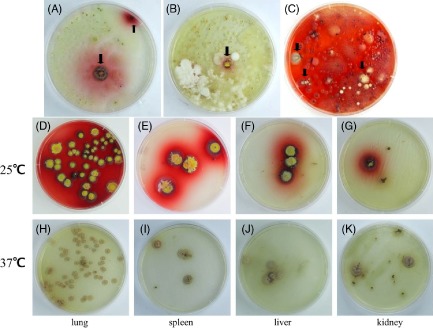
The isolation of *P. marneffei* recovered from different samples. Black arrows point to the suspected colonies. (A-C) Growth of *P. marneffei* recovered from soil, bamboo root and stool of bamboo rats at 25°C. (D-K) The growth of *P. marneffei* recovered from different organs of bamboo rats at 25°C and 37°C.

Among the total 184 samples obtained from the natural environment of Guangdong province, the isolated rate of *P. marneffei* was 5/41 in soil, 4/37 in root, 0/27 in petiole, 0/40 in leaves, 0/13 in debris of food and 6/27 in stool respectively (Table 2). Most of the positive isolates were recovered from different areas. As data showed, *P. marneffei* can be isolated from the soil and the root nearby the burrow, but was not recovered in the petiole, the leaves and the debris of food from the surrounding areas (Fig. 2A–C).

**Table 2:** The positive ratio of *P. marneffei* recovered from different samples, which collected from areas surrounding burrows

Area	Samples					
	Soil	Root	Petiole	Leaves	Debris of food	Stool
Chaozhou	1/6	1/6	–	0/7	–	2/4
Qingyuan	2/6	0/6	0/4	0/6	0/4	0/2
Zhaoqing	1/8	1/8	0/4	0/8	–	2/6
Meizhou	0/10	2/8	0/8	0/8	0/2	1/8
Shaoguan	1/11	0/9	0/10	0/11	0/7	1/7
Total	5/41	4/37	0/26	0/40	0/13	6/27

A total of 36 samples, which included nine specimens were collected from the soil, the root, the leaves and the debris of food respectively, were obtained from the artificial propagation farm of bamboo rat. None of these samples were positive for *P. marneffei*.

### The isolation of P. marneffei in bamboo rats

**Fig 3:**
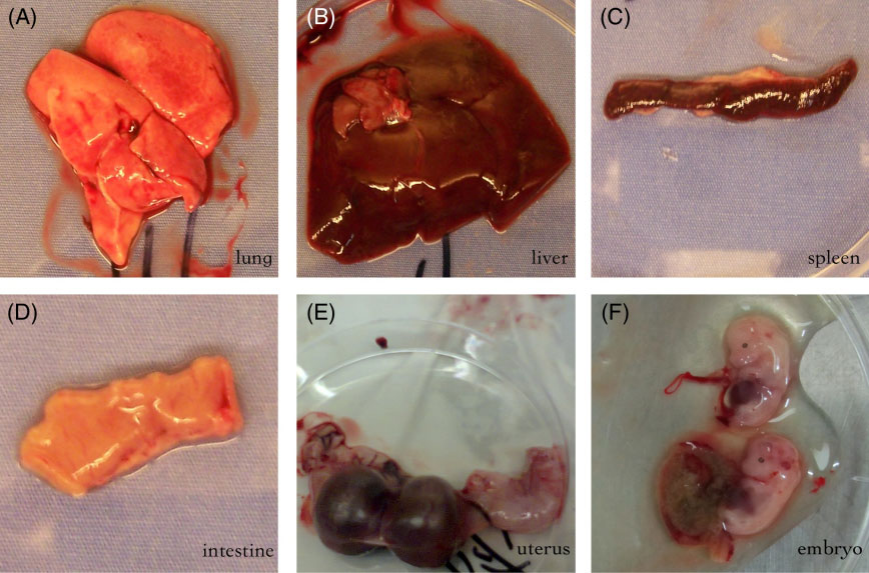
The organs of bamboo rat we captured. (A) Lung; (B) liver; (C) spleen; (D) intestine; (E) uterus; (F) embryo.

All six bamboo rats captured in investigated sites were positive for *P. marneffei*. The bamboo rats appeared healthy, and at necropsy, no visible pathologic changes were observed on any of the internal organs of the rats. Figure 3 revealed the organs taken from the captured bamboo rats. In all six bamboo rats, *P. marneffei* was cultured from lungs. Cultures of the liver and the spleen from five rats yielded *P. marneffei*. However, only one kidney tissue culture produced *P. marneffei*. None of the cultures from the intestine, lymph node, and embryo or amniotic fluid yielded the fungus (Table 3). Figure 2D–K shows that the colonies of *P. marneffei* isolated from the lungs, the liver, the spleen and the kidney grew at 25°C and 37°C with a characteristic red diffused pigment at 25°C and growing as yeast form at 37°C.

**Table 3:** The positive ratio of *P. marneffei* recovered from different organs of bamboo rat

Areas	Visceral organs								
	Lung	Liver	Spleen	Kidney	Intestines	Intestinal contents	Lymph node	Embryo	Amniotic fluid
Chaozhou	1/1	1/1	1/1	0/1	–	–	–	N/A	N/A
Qingyuan	1/1	1/1	1/1	0/1	0/1	0/2	–	N/A	N/A
Zhaoqing	2/2	1/2	1/2	0/2	0/2	0/2	0/1	N/A	N/A
Meizhou	1/1	1/1	1/1	1/1	0/1	0/3	0/1	N/A	N/A
Shaoguan	1/1	1/1	1/1	0/1	0/1	–	–	0/1	0/1
Total	6/6	5/6	5/6	1/6	0/5	0/7	0/2	0/1	0/1

## Discussion

In this investigation, we selected six different areas in the natural environment, located in the east, west, middle and north of Guangdong province. A total of 270 samples were collected and 16 samples among them were positive for *P. marneffei*. Earlier studies have provided scarce information about the environmental prevalence of *P. marneffei*. In Thailand, Chariyalertsak and colleagues (1996a) cultured only one positive isolate from 28 soil samples around the bamboo rat burrows in 1996. In India, Gugnani and colleagues (2004; 2007) failed to recover *P. marneffei* from the burrows of bamboo rat (*Cannomys badius*). In Mainland China, only Deng and colleagues (1986) succeeded in culturing this fungus from three soil samples in wild area of Guangxi province in 1987. To our knowledge, this study demonstrated high numbers of positive isolates of *P. marneffei* from natural sources for the first time.

Some previous studies have attempted to find epidemiological link between bamboo rats and human infection, but till now it showed little association (Chariyalertsak *et al*., 1996a; 1997). As *P. marneffei* infection is thought to be a sapronosis, this fungus must have a reservoir in the environment. So it would be meaningful to explore the distribution of *P. marneffei* surrounding the habitat of bamboo rats. We observed that the environmental samples collected from soil, plant roots and stool have relatively high isolation rates, while no isolate was cultured from petiole, leaves and the debris of food. We hypothesized that soil, plant roots and stool are suitable for harvest of *P. marneffei*, or they contain certain substances, which are necessary for the survival of this fungus. Although further investigations are required to confirm it, this study has provided definitive proof of an environmental reservoir for *P. marneffei* within the soil, plant roots and stool.

Our previous studies have collected wild rats, including *Microtu*s, *focus Rattus* and *Rhizomys pruinosus*, for investigating the natural host of *P. marneffei*, and found only bamboo rats were positive for *P. marneffei* (Li *et al*., 2011,2011). It revealed that there are host-specific factors that govern *P. marneffei* infection. Geographical variation in the predisposition to infection within different species of bamboo rats might exist. In Mainland China, Li and colleagues (2011,2011) and Deng and colleagues (1986) have revealed that infection in *R. pruinosus* has a high prevalence in Guangdong and Guangxi Province, while infection in *R. sumatrensis* and *C. badius* showed higher prevalence in northern Thailand (Chariyalertsak *et al*., 1996a) and India (Gugnani *et al*., 2004). The same situation might have occurred in surrounding area of rats' burrows. Till now, including our study, only the samples collected from burrows of *R. sumatrensis* and *R. pruinosus* showed positive for *P. marneffei* (Deng *et al*., 1986; Chariyalertsak *et al*., 1996a).

We trapped six bamboo rats (*R. pruinosus*) from five sites across a wide geographic region in Guangdong province, and revealed that infection is prevalent at 100% in all rats. Among the internal organs of these rats, the strains were most frequently isolated from lungs, which were consistent with our previous studies (13/23 in lungs) (Li *et al*., 2011,2011). However, no isolate was recovered from the embryonic tissue of pregnant rats. Cao and colleagues also showed that no isolate was recovered from the embryonic tissue of pregnant rats (*n* = 15) (Cao *et al*., 2011). Moreover, no isolate was recovered from debris of food, intestines tissue and intestinal contents. All these results support our previous hypothesis that bamboo rats become infected by inhaling aerosolized conidia originating from environmental sources (Li *et al*., 2011,2011), rather than by fecal–oral route or transplacental crossing.

Above all, this study provided definitive evidence for natural occurrence of *P. marneffei* in natural sources, including soil and plant roots. Humans and bamboo rats may acquire this infection from a common soil reservoir, but it is unclear whether the infection occurs from soil exposure because *P. marneffei* infection occurred both in rural and urban populations. It is interesting that the prevalence of *P. marneffei* was much higher in samples collected from surrounding areas of burrows (8.2%) than in the samples obtained from non-rat-associated sites (2%) or artificial farms of bamboo rats (0%). A possible explanation is that the bamboo rats in artificial propagation farm have limited range of activities. Other factors may also be associated with an increased risk of infection of isolation. Studies in Thailand (Chariyalertsak *et al*., 1997) and Vietnam (Bulterys *et al*., 2013) found that more *P. marneffei* infections develop during a rainy season. Also, Bulterys and colleagues (Bulterys *et al*., 2013) found that *P. marneffei* hospital admissions were strongly associated with humidity, while precipitation, temperature and wind did not add explanatory power. These may suggest that humidity or rain facilitate *P. marneffei* growing on air-exposed plant and soil surfaces, which may serve as a crucial step in the infection of bamboo rats and immunocompromised human.

## Experimental procedures

### Description of study area

**Fig 4:**
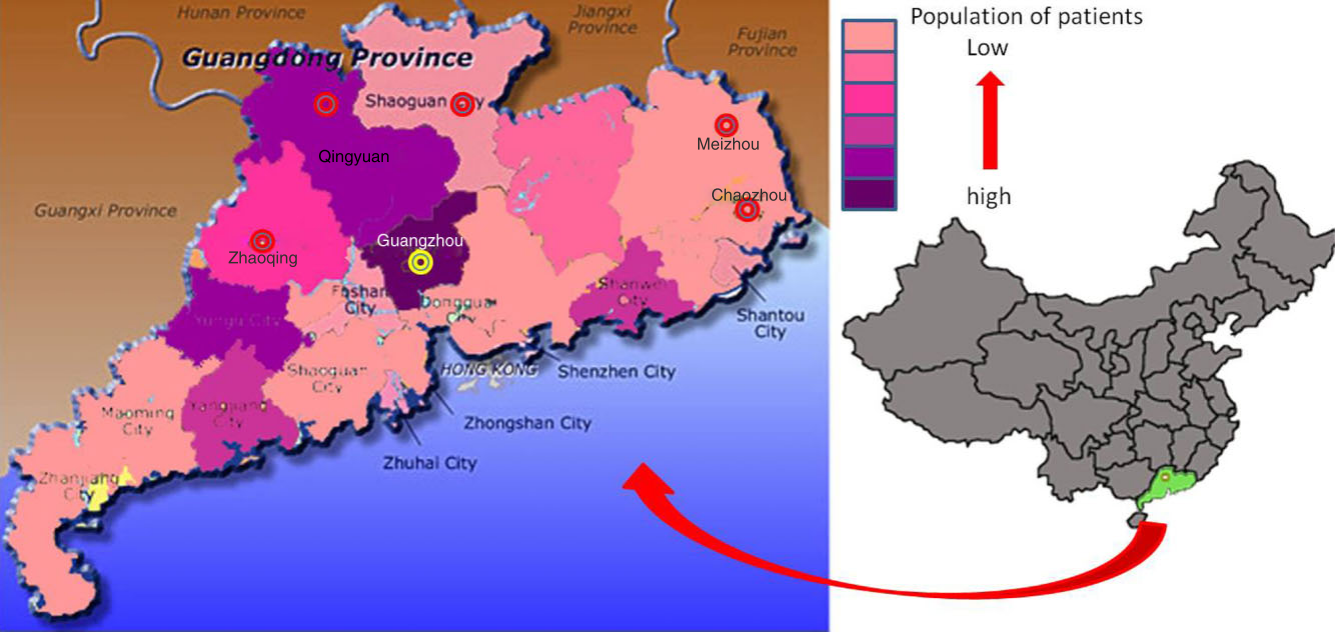
Map of Guangdong Province of China, showing population of penicilliosis marneffei patients and the locations of Chaozhou, Qingyuan, Zhaoqing, Meizhou and Shaoguan city where samples were collected (red circle represents districts for collecting environmental samples, yellow circle represents district of artificial farm of bamboo rats).

All isolates were collected according to the data, which showed the high population of patients with penicilliosis marneffei in Guangdong province (Xi *et al*., 2004; Li *et al*., 2011,2011). Guangdong province situates from 20.12°N to 25.31°N and from 109.45°E to 117.20°E. The region has a subtropical humid climate, with temperatures ranging between 18°C and 35°C in summer. The rainy season lasts from April to September. The sites where bamboo rats were trapped are located in Chaozhou, Qingyuan, Zhaoqing, Meizhou and Shaoguan (Fig. 4). All of these sites are predominantly hills state with an elevation ranging from 500 to 1900 m above sea level.

### Investigation of samples

All samples were collected from May to September. Farmers trapped six *R. pruinosus* (five male and one female) from five districts (Fig. 4). The bamboo rats were taken to the laboratory within 2–3 days. Portions of lungs, kidney, liver, spleen and embryos (Fig. 3) were minced into tiny pieces aseptically and then incubated on Sabouraud dextrose agar (SDA) plates with 40 μg ml^–1^ gentamicin. Cultures were incubated both at 25°C and 37°C for 3–4 weeks.

The soil, root, petiole, leaves, debris of food and stool samples were collected from the burrows of bamboo rats. Meanwhile, 50 soil samples were also collected from the place far away from each burrow. Thirty-six samples were collected from propagation farm of bamboo rats. This artificial propagation farm is located in rural region of Guangzhou, which is located in the middle of Guangdong province, and it utilizes the captive method to breed bamboo rats (*R. pruinosus*). All samples were transported back to laboratory in sterile plastic containers.

Three grams of each sample were suspended in 10 ml of sterile distilled water (SDW) with 80 μg ml^–1^ gentamicin. The soil suspension was mixed vigorously for 5 min. After settling for 20 min, 1 ml of the suspension was added to 9 ml of SDW to get 1:10 and 1:100 dilutions. The prepared samples (0.2 ml) were streaked onto SDA plates with 40 μg ml^–1^ gentamicin. All the inoculated plates were incubated at 25°C for 3–4 weeks.

### Isolate identification

The colonies with red pigment were purified by subculture for identification. The isolates were further identified based on their gross and microscopic morphology (Gugnani *et al*., 2007). Isolates from these cultures were incubated on SDA at 37°C to examine for dimorphism. DNA was extracted using the InStaGene Matrix (Bio-Rad, USA) according to the manufacturer's instructions. A sequence analysis of the entire internal transcribed spacer region (ITS4 and ITS5 primers were used to amplify the entire ITS region) was used to confirm the *P. marneffei* strains (Li *et al*., 2011,2011).

## Conflict of interest

The authors declare that they have no conflicts of interests.
